# Cryoablation: A Minimally Invasive Alternative for Early-Stage Breast Cancer: 6-Year Outcomes of the FROST Clinical Trial

**DOI:** 10.1245/s10434-025-18991-2

**Published:** 2026-01-15

**Authors:** D. R. Holmes, S. Manoian, R. Layeequr Rahman, R. C. Ward, N. Z. Carp, M. Plaza, K. Kozlowski, S. Abe, L. Bailey, L. Kruper, V. Jones, S. Patterson, J. Tamayo, P. Littrup

**Affiliations:** 1 Adventist Health Glendale, Glendale, CA USA; 2 90120 Surgery Medical Center, Beverly Hills, CA USA; 3 Texas Tech University Health Sciences Center, Lubbock, TX USA; 4 Southwest Cancer Center, Lubbock, TX USA; 5 Brown University Warren Alpert Medical School, Providence, RI USA; 6 Lankenau Medical Center, Wynnewood, PA USA; 7 Hampton Roads Radiology Associates, Virginia Beach, VA USA; 8 Diagnostic Center for Women, Miami, FL USA; 9 Knoxville Comprehensive Breast Center, Knoxville, TN USA; 10 University of California San Francisco, San Francisco, CA USA; 11 Epic Care, Emeryville, CA USA; 12 Alta Bates Summit Medical Center, Oakland, CA USA; 13 Moffitt Cancer Center, Tampa, FL USA; 14 City of Hope Comprehensive Cancer Center, Duarte, CA USA; 15 Magnolia Breast Center, Naples, FL USA; 16 Naples Community Hospital, Naples, FL USA; 17 Ogden Regional Medical Center, Ogden, USA; 18 University of Rochester Medical Center, Rochester, USA; 19 Ascension Crittenton Hospital, Rochester, MI USA

**Keywords:** FROST, Cryoablation, Breast cancer, Ablation, Minimally invasive, Cryoprobe

## Abstract

**Background:**

Cryoablation is emerging as a minimally invasive alternative to lumpectomy for select women with early-stage breast cancer. The FROST trial (NCT01992250) was a prospective, phase 2 multicenter study evaluating the outcome of cryoablation in the management of stage I, hormone receptor-positive, human epidermal growth factor receptor 2 (HER2)-negative, node-negative invasive ductal carcinoma.

**Methods:**

Women 50 years old or older with unifocal, ultrasound-visible tumors were stratified by age: stratum 1 (age ≥70 years, endocrine therapy only) and stratum 2 (age 50–69 years, endocrine therapy + radiotherapy + optional sentinel node biopsy). Cryoablation was performed using a single cryoprobe under ultrasound guidance. Core biopsy 6 months after ablation was performed to confirm complete ablation. Patients were followed with clinical exams and imaging.

**Results:**

The study included 83 completed cryoablations and follow-up evaluations. The median tumor size was 9 mm. More than 85% of the subjects in each group received endocrine therapy (stratum 1 [89%, 43/48], stratum 2 [85.7%, 30/35]) and 74.3% (26/35) of the subjects in stratum 2 received recommended whole-breast radiation. Of the 83 patients, 82 received a post-ablation core biopsy 6 months after cryoablation showing no residual cancer, and 1 patient declined a core biopsy. During a median follow-up period of 6.1 years, the 5-year ipsilateral breast tumor recurrence rate (IBTR) was 3.64% overall (stratum 1, 2.08%; stratum 2, 5.80%). The invasive IBTR-free survival rate was 97.59% overall (stratum 1, 97.92%; stratum 2, 97.14%). No serious adverse events occurred.

**Conclusions:**

The FROST trial adds to the growing body of literature supporting the efficacy and safety of cryoablation and supports ongoing research on cryoablation as a strategy for de-escalating breast cancer therapy.

**Supplementary Information:**

The online version contains supplementary material available at 10.1245/s10434-025-18991-2.

Breast-conserving surgery remains the standard of care for early-stage breast cancer. However, the increasing shift toward de-escalated and patient-centered care has fostered growing interest in minimally invasive alternatives that may reduce the physical and psychological burden of surgery. Advances in high-resolution imaging, together with a more nuanced understanding of tumor biology, currently provide the basis for identifying low- and intermediate-risk patients who may benefit from less invasive interventions.

Cryoablation is a percutaneous thermal ablative technique gaining attention as an alternative to surgery for early-stage breast cancer.^1^ Unlike heat-based ablation methods, such as radiofrequency or microwave ablation, which typically require sedation or anesthesia, cryoablation induces analgesia via tissue freezing. This characteristic enables the procedure to be performed with the patient under local anesthesia in an office or clinic setting (Supplemental Image 1).^2,3^

Cryoablation has become a standard treatment option for kidney, liver, and prostate cancers, and is an emerging option for treatment of breast cancer.^4–7^ The ICE3 trial, the largest published prospective study of cryoablation for early-stage breast cancer, demonstrated that office-based cryoablation without surgery was safe and well-tolerated, achieving a 5-year ipsilateral breast tumor recurrence (IBTR) rate of only 4.3% for appropriately selected patients.^8^ The current study reports the 6-year outcomes of the Freezing Instead of Resection of Small Tumors (FROST) trial, a prospective study evaluating cryoablation as definitive local therapy for a broader population of women with early-stage breast cancer.

## Materials and Methods

The FROST trial was a phase 2, prospective, non-randomized study evaluating cryoablation as primary treatment for early-stage breast cancer. Eligible patients were women 50 years old or older with ultrasound-visible, unifocal, clinical stage I (T1, ≤2.0 cm), hormone receptor-positive, human epidermal growth factor receptor 2 (HER2)-negative invasive ductal carcinoma confirmed by core biopsy, with no evidence of axillary adenopathy by physical examination and axillary ultrasound. All patients received diagnostic mammography. Contrast-enhanced magnetic resonance imaging (CE-MRI) was optional. The exclusion criteria ruled out non–ultrasound-visible tumors, extensive ductal carcinoma *in situ* (DCIS ≥25% of core biopsy), multifocal or multicentric disease, prior ipsilateral cancer, and neoadjuvant therapy. The study was institutional review board (IRB)-approved and listed on ClinicalTrials.gov (NCT01992250). All cases were centrally reviewed by the study PI to confirm that eligibility criteria were met.

The primary trial objective was to determine the rate of successful tumor ablation (i.e., response rate) among subjects treated with cryoablation of the primary tumor instead of surgical resection. Successful tumor ablation was defined as absence of residual viable invasive carcinoma or DCIS detected by core biopsy of the cryoablation zone 6 months after cryoablation.

The secondary outcome was the 5-year IBTR rate, with IBTR defined by the trial to be the reappearance of invasive or non-invasive breast cancer within the same breast ≤5 cm from the original tumor site, as measured from the location of the original biopsy site marker. Additional secondary outcomes included invasive breast tumor recurrence-free survival (IBTR-FS), overall survival (OS), and incidence of adverse events.

Clinical records were systematically reviewed to identify any post-procedural complications, describe their clinical management, and determine whether the events were attributable to the cryoablation procedure, breast radiation, or endocrine therapy. The IBTR after cryoablation was evaluated using clinical examination and diagnostic imaging, including mammography, breast ultrasound, CE-MRI (optional), and core needle biopsy, if indicated. Kaplan–Meier survival analysis was used to estimate the 5-year IBTR rate, 5-year invasive IBTR-FS rate, and 5-year OS rate, with corresponding 95% confidence intervals (CIs).

### Patient Strata

**Fig. 1:**
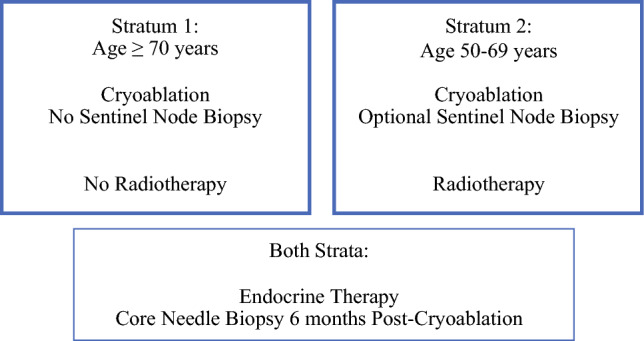
Treatment protocol for two age-based strata.

The FROST trial was designed as a risk-adapted study, with participants stratified into two age-based cohorts guided by widely accepted clinical criteria for loco-regional recurrence risk (Fig. 1). Stratum 1 included women 70 years old or older with stage I (T1 $\le$ 2.0 cm), hormone receptor-positive, HER2-negative invasive ductal carcinoma. These criteria mirrored the CALGB 9343 clinical trial, a randomized controlled trial comparing lumpectomy, tamoxifen, and whole-breast radiation with lumpectomy and tamoxifen without whole-breast radiation in which only one third of the participants received optional sentinel node biopsy or axillary node dissection. The CALGB 9343 trial demonstrated no OS benefit from adding radiation to endocrine therapy for women 70 years old or older treated with lumpectomy and optional axillary surgery.^8^ In the FROST clinical trial, the women in stratum 1 received cryoablation and also were prescribed a 5-year course of endocrine therapy. Lymph node surgery and radiation were not routinely performed. Stratum 2 included women 50–69 years of age with similar tumors. Due to higher recurrence risk in this age group, all the stratum 2 participants were prescribed adjuvant radiotherapy in addition to cryoablation and endocrine therapy. Lymph node surgery was optional.

### The Cryoablation Procedure

**Fig. 2:**
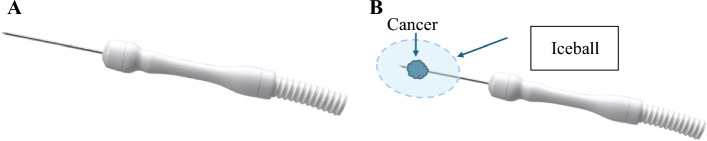
**A** 10-Gauge disposable cryoprobe and handpiece. **B** Cryoprobe inserted through center of mass with surrounding iceball

Cryoablation was performed in-office or in radiology departments with the Visica2 Cryoablation System (Sanarus Medical, Inc., Pleasanton, CA, USA), using liquid nitrogen and a single 10-gauge cryoprobe (Fig. 2A). The cryoablation procedures were performed by 14 different providers. All the providers were either breast surgeons experienced with cryoablation or radiologists with expertise in breast imaging and biopsies. Device company representatives were present for one or more cases based on provider experience performing cryoablation.

With the patient under local anesthesia, a 3-mm skin incision was made, and the cryoprobe was inserted into the tumor center under ultrasound guidance (Fig. 2B). Two freeze cycles were applied, separated by a 10-min passive thaw. For tumors smaller than 1 cm, each freeze cycle was 6 min, and for tumors 1–2 cm, each cycle was 10 min. However, freezing was to be continued until the iceball perimeter extended 10 mm beyond tumor margins (Supplemental Image 2). Only two patients (one in each stratum) required prolongation of freeze time to achieve iceball margins $\ge$ 10 mm. Saline was injected subcutaneously as needed to displace skin from the iceball to prevent skin injury. After the final freeze, the cryoprobe was actively warmed and removed. The total procedure time averaged less than 60 min.

### Sentinel Node Biopsy

Sentinel node biopsy was optional in stratum 2. The protocol suggested but did not mandate the use of genomic testing to identify biologically high-risk patients who might benefit from sentinel node biopsy, chemotherapy, or both. Sentinel node biopsy was not performed for the patients enrolled in stratum 1.

### Adjuvant Radiotherapy

The patients in stratum 2 generally initiated external beam radiotherapy to the whole breast and lower axilla 2–4 months after cryoablation. The radiation treatment regimen was left to the discretion of the treating radiation oncologist.

### Adjunctive Systemic Therapy

Adjuvant endocrine therapy was started either immediately after cryoablation (stratum 1) or after the completion of radiotherapy (stratum 2). Premenopausal women were prescribed tamoxifen, whereas postmenopausal women were generally prescribed aromatase inhibitors. Medication adherence was monitored at follow-up visits as reported by the patients.

### Follow-Up Protocol

Post-cryoablation follow-up visits and breast examinations were scheduled at 2 weeks, 1 month, 3 months, and every 6 months for 5 years. Imaging included annual mammography, ultrasound, and optional CE-MRI. Adverse events were assessed and graded using Common Terminology Criteria for Adverse Events (CTCAE) v4.03.^9^ At 6 months post-procedure, the study participants underwent core needle biopsy of the cryoablation site using a standard biopsy protocol targeting the central aspect of the cryoablation zone or residual tumor scar if visible.

### Sample Size

The study used Simon’s two-stage design.^10^ If fewer than $22$ of the first 42 patients per stratum had successful ablation (confirmed by 6-month post-cryoablation biopsy), enrollment would stop. Otherwise, accrual would continue to 105 patients per stratum. This design offered 90% power with a 5% type 1 error to detect at a 65% response rate. The target enrollment goal was 231 patients to account for dropouts. However, the target enrollment was not met due to premature study closure resulting from acquisition of the trial’s industry sponsor by another company that elected not to continue the study. Consequently, we were unable to achieve full patient accrual within each study arm. Nevertheless, despite the loss of funding, co-investigators volunteered their time to provide long-term follow-up evaluation for the study participants, allowing us to report these important long-term results.

## Results

**Fig. 3:**
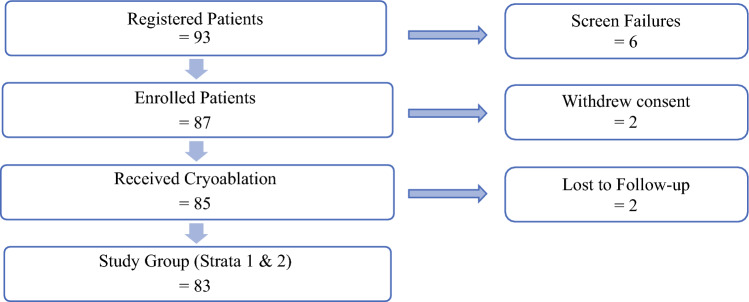
Patient screening and enrollment

Between 22 July 2016, and 21 October 2020, 93 patients registered at 12 U.S. sites (Supplemental Image 3). Six patients failed screening; two withdrew consent; and two were lost to follow-up evaluation, leaving 83 evaluable patients: 48 in stratum 1 and 35 in stratum 2 (Fig. 3). The combined cohort was 79.1% white, 9.3% black, 5.8% Hispanic, and 5.8% Asian (Table 1). The median age was 71 years overall (76 years in stratum 1 and 62 years in stratum 2).

**Table 1:** Characteristics of enrolled patients

Race/ethnicity	Stratum 1 (%)	Stratum 2 (%)	Total (%)
White	79.1	77.1	79.1
Black	6.3	14.3	9.3
Hispanic	6.3	5.7	5.8
Asian	8.3	2.9	5.8
Age			
Mean years	76 ± 4	62 ± 5	70 ± 9
Median years (range)	76 (70–95)	62 (51–69)	71(51–95)
Histology			
Infiltrating ductal carcinoma	48 (100)	35 (100)	83 (100)
Grade			
Low	44.9	37.2	41.1
Intermediate	53.1	60.5	56.8
High	2.0	2.3	2.1
Hormone receptor status			
ER+/PR+	95.9	97.1	96.5
ER+/PR–	4.1	2.9	3.6
ER–/PR+	0	0	0
HER2–	100	100	100
Tumor size by ultrasound			
Median: mm (range)	9.5 (4–19)	8.0 (4–18)	9.0 (4–19)
Mean	9.6 ± 3.2	9.0 ± 3.1	9.3 ± 3.2

ER, estrogen receptor; PR, progesterone receptor; HER2, human epidermal growth factor receptor 2

All tumors were hormone receptor-positive, HER2-negative invasive ductal carcinomas, as confirmed by diagnostic core biopsy (Table 1). Histologic grading showed a predominance of intermediate-grade tumors (56.8%), followed by low-grade (41.1%) and high-grade (2.1%) tumors. The median maximum tumor diameter was 9.0 mm (range: 4–19 mm) across the entire cohort: 9.5 mm (range: 4–19 mm) in stratum 1 and 8.0 mm (range: 4–18 mm) in stratum 2. Although breast CE-MRI was optional, all but one patient underwent breast CE-MRI before cryoablation. The data cutoff date for all subjects was 31 October 2024. At that time, the median follow-up duration was 6.11 years for the overall study population: 5.91 years for stratum 1 and 6.38 years for stratum 2 (Table 2).

**Table 2:** Mean and median length of follow-up period in years for stratum 1, stratum 2, and both strata combined

Variable	Stratum= Stratum 1 (N=48)	Stratum= Stratum 2 (N=35)	Stratum=Total (N=83)
Follow-Up Time in years			
Mean (SD)	5.91 (1.18)	6.38 (1.03)	6.11 (1.14)
Median (Q1, Q3)	6.13 (5.38, 6.61)	6.52 (5.92, 7.16)	6.23 (5.52, 6.87)
Min, Max	1.98, 7.80	3.66, 8.28	1.98, 8.28

During follow-up evaluation, 89.6% (44/48) of the participants in stratum 1 received a minimum of 4 years of adjuvant endocrine therapy. In stratum 2, 71.4% (25/35) of the patients received endocrine therapy for 4 years or longer, 74.3% (26/35) received recommended whole-breast radiation, and 5.7% (2/35) declined both endocrine therapy and radiotherapy. Genomic testing was documented for 28.5% (10/35) of the patients in stratum 2, with a high-risk score reported for only one individual, who subsequently refused chemotherapy. Sentinel node biopsy was reported for only 11.4% (4/35) of the patients in stratum 2, with no metastatic carcinoma identified. No patients received chemotherapy.

### Response Rate

Most patients in both strata (82/83; 98.8%) underwent ultrasound-guided core biopsies of the cryoablation site 6 months after cryoablation. Histopathologic examination of the core biopsy specimens typically showed fibrosis, fat necrosis, and/or chronic inflammatory changes—expected findings after tissue ablation. Importantly, no cases of residual invasive carcinoma or DCIS were identified at the original tumor site or within the cryoablation zone. The one patient who declined core biopsy was a 94-year-old patient in stratum 1 treated during the COVID-19 pandemic.

### Ipsilateral Breast Tumor Recurrence (IBTR)

A total of three study-defined IBTRs were documented during the follow-up period, yielding a 5-year IBTR rate of 3.64% overall (stratum 1, 2.08%; stratum 2, 5.80%) by Kaplan-Meier estimates (Table 3). One of the recurrences occurred in a stratum 1 patient who had undergone cryoablation at age 70 years for a 11-mm intermediate-grade invasive ductal carcinoma. The patient had initiated adjuvant endocrine therapy but experienced an 8.5-mm intermediate-grade invasive ductal carcinoma 12 months later, located just beyond the margins of the cryoablation zone. The recurrence was subsequently managed with lumpectomy.

**Table 3:** Mean 5-year Kaplan-Meier IBTR rates and confidence intervals for individual strata and combined strata

	Stratum= Stratum 1 (N=48)	Stratum= Stratum 2 (N=35)	Stratum=Total (N=83)
5-Year Failure Rate	2.08%	5.80%	3.64%
Lower CI	0.00%	0.00%	0.00%
Upper CI	6.04%	13.29%	7.61%

IBTR, ipsilateral breast tumor recurrence

A second IBTR occurred in a patient who underwent cryoablation at age 59 years (stratum 2) for a 17-mm intermediate-grade invasive ductal carcinoma. After cryoablation, the patient declined endocrine therapy and radiotherapy. At 12 months after cryoablation, a 10-mm intermediate-grade invasive ductal carcinoma was identified within the index quadrant 2 cm from the original cryoablation zone. The recurrence was ultimately managed with lumpectomy.

A third IBTR recurrence occurred in a patient who underwent cryoablation at age 60 years (stratum 2) for an 11-mm low-grade invasive ductal carcinoma. The patient had initiated endocrine therapy but had declined recommended radiotherapy. At 5 years after cryoablation, a 5 mm intermediate-grade invasive ductal carcinoma was detected in the index quadrant outside the original cryoablation zone. The recurrence was ultimately managed with lumpectomy followed by radiotherapy.

A fourth IBTR occurred more than 5 cm from the index lesion, resulting in an overall whole-breast IBTR rate of 4.8%. However, because this event did not meet the FROST trial’s predefined criteria for an IBTR (i.e., ≤5 from the original tumor site), it was excluded from the IBTR and IBTR-FS rate calculations. The patient underwent cryoablation at age 60 years (stratum 2) for a 14-mm intermediate-grade invasive ductal carcinoma but declined adjuvant endocrine therapy and radiotherapy. At 2.2 years after cryoablation, a 10-mm intermediate-grade invasive ductal carcinoma was detected 6.4 cm from the original tumor site. The patient was ultimately treated with mastectomy.

Two patients experienced ipsilateral axillary recurrences. The first patient, age 60 years (stratum 2), underwent cryoablation for a 15-mm intermediate-grade invasive ductal carcinoma, initiated endocrine therapy, but declined adjuvant radiotherapy. The second patient, age 76 years (stratum 1), underwent cryoablation for a 13-mm intermediate-grade invasive ductal carcinoma, initiated endocrine therapy, but experienced an ipsilateral axillary recurrence within 6 months. Both patients were subsequently treated with an axillary lymph node dissection and radiotherapy.

### Invasive Ipsilateral Breast Tumor Recurrence–Free Survival (IBTR-FS)

Among the three documented IBTRs, one was a case of ductal carcinoma *in situ* (DCIS), leaving two invasive IBTRs (one in each stratum) for the invasive IBTR-FS rate calculation. Based on Kaplan–Meier estimates, the 5-year invasive IBTR-FS rate was 97.59% (95% CI, 94.35–100.00%) for the overall study population, 97.92% (95% CI: 93.96–100.00%) for stratum 1, and 97.14% (95% CI: 91.78–100.00%) for stratum 2 (Table 4).

**Table 4:** Mean 5-year Kaplan-Meier invasive IBTR-FS rates for individual strata and combined strata

	Stratum= Stratum 1 (N=48)	Stratum= Stratum 2 (N=35)	Stratum=Total (N=83)
5-Year Local Recurrence Free Survial Rate	97.92%	97.14%	97.59%
Lower CI	93.96%	91.78%	94.35%
Upper CI	100.00%	100.00%	100.00%

IBTR-FS, ipsilateral breast tumor recurrence-free survival

### Overall Survival

Three non–breast cancer-related deaths were reported during the follow-up period: two in stratum 1 and one in stratum 2. Importantly, no breast cancer-related deaths occurred in either cohort. Based on Kaplan–Meier estimates, the 5-year OS rate was 96.36% for the combined strata, 97.92% for stratum 1, and 94.20% for stratum 2.

These high 5-year OS rates (94–98%) across both strata suggest excellent disease control and favorable clinical outcomes. However, the relatively wide confidence intervals, particularly in stratum 2, reflect the low number of observed mortality events, which limits the statistical precision of the survival estimates.

### Adverse Events

Adverse events were assessed in accordance with CTCAE v4.03.^9^ No serious cryoablation or radiation-related adverse events were observed. Mild-to-moderate (grades 1**–**2) pain, bruising, and skin erythema were commonly reported in the first 2 weeks after cryoablation. One patient experienced a grade 2 axillary infection, which was resolved with oral antibiotics. Supplemental Image 4 summarizes the adverse events reported 2 weeks and 6 months after ablation.

### Statistical Power Analysis

Premature study closure also raised concerns about the significance of the study’s findings. In stratum 1, 47 (97.9%) of 48 patients achieved complete ablation. The critical value was a *k* of 31, with a type 1 error tail probability of 0.0297 at a *p*0<AQ4> value of 0.5. The one-sided *p* value was extremely small (<0.0001), and the post hoc power was essentially 100%. In stratum 2, 35 (100%) of 35 patients achieved complete ablation. The critical value was a *k* of 23, with a type 1 error tail probability of 0.0448. Again, the one-sided *p* value was lower than 0.0001, and the post hoc power was 100%.

These findings are consistent with the very low incidence of IBTR observed, only three events altogether, supporting the durability of local control. Although the study was not originally powered for IBTR outcomes, the results nonetheless demonstrate high 5-year IBTR-FS rates with sufficient statistical strength to exceed clinically relevant benchmarks. Notably, both stratum 1 and the overall cohort achieved survival rates exceeding 80%, and in some cases even 90 % (Supplement–Power Analysis).

## Discussion

Cryoablation is emerging as an alternative to surgery for the management of early-stage breast cancer. The FROST trial documented the short-term efficacy of cryoablation, as evidenced by the absence of residual cancer assessed by core needle biopsy of the cryoablation site 6 months after cryoablation. The 6-year follow-up evaluation of the FROST trial adds to the body of evidence presented by the ICE3 trial, which is the largest published prospective clinical study assessing the long-term results of breast cancer cryoablation for early-stage disease.^8^ Together, these trials demonstrate promising local control rates, low recurrence, and favorable safety profiles, supporting cryoablation as a minimally invasive treatment option for appropriately selected patients with early-stage breast cancer.

Despite similar inclusion criteria, key distinctions between the two trials may support expanding the eligibility criteria for cryoablation. The ICE3 trial enrolled women age $\ge$ 60 years (median, 74.5 years; range, 55–94 years) with low-risk, stage I, hormone receptor-positive, HER2-negative invasive breast cancer. On the other hand, the FROST trial was intentionally designed as a risk-adapted study to evaluate the safety and efficacy of cryoablation in younger patients, lowering the age threshold to 50 years to extend access to this minimally invasive treatment. Notwithstanding the FROST trial’s broader inclusion criteria, the FROST trial’s 5-year IBTR rate (3.6%, study-defined) and the 5-year IBTR rate (4.8%, whole-breast) are comparable with the 4.3% 5-year IBTR rate reported in the ICE3 trial.

Similar to stratum 1 in the FROST trial, relatively few (13.9%) ICE3 trial participants received adjuvant radiotherapy, reflecting evolving clinical practice guidelines that support omitting radiation for older patients. This approach has been validated by several prospective trials, including the CALGB 9343, PRIME II, LUMINA, and IDEA trials.^8,11–13^ Although the omission of radiation therapy after breast conservation procedures had already become a widely accepted practice by 2016 when the FROST and ICE3 trials were designed, only recently have clinical trials provided high-level evidence supporting the selective omission of radiation therapy after breast-conserving surgery for women as young as 50 years (Table 5).^4,8,11–14^

**Table 5:** Comparison between 5-year IBTR reported in contemporary clinical trials evaluating the efficacy of omitting adjuvant radiotherapy after breast-conserving lumpectomy (IDEA, LUMINA, CALGB 9343, PRIME II, ABCSG) and cryoablation (FROST, ICE3)

Trial	Age criteria (years)	Median age (years)	Median tumor size (mm)	5-Year IBTR rate
IDEA	50–69	66	11	1.3
FROST stratum 1	$\ge 70$	76	9.5	2.1
LUMINA	$\ge 55$	67	11	2.3
CALGB 9343	$\ge 70$	77	11	4.0
PRIME II	$\ge 65$	76	13	4.1
ICE3	$\ge 60$	75	9	4.3
ABCSG	$\ge$ 50	66	12	5.1

IBTR, ipsilateral breast tumor recurrence

Both the FROST and ICE3 trials were designed and conducted during a pivotal era in which the breast and surgical oncology communities began reevaluating long-standing dogma regarding the necessity of surgical axillary staging in both early-stage and advanced breast cancer. The fact that only 7.7% of the ICE3 participants underwent sentinel lymph node biopsy reflects alignment with evolving standards of care, particularly the Society of Surgical Oncology’s *Choosing Wisely* recommendations, which advise against routine sentinel node biopsy for women age 70 years or older with stage I, clinically node-negative, hormone receptor-positive, HER2-negative invasive breast cancer who are willing to take endocrine therapy.^15^ The same principles informed the decision to omit routine sentinel node biopsy in stratum 1 of the FROST trial.

To further support de-escalation of surgical intervention in a population undergoing a primarily office-based procedure, the FROST trial also permitted optional sentinel node biopsy in stratum 2. This flexible, risk-adapted approach allowed clinicians to tailor axillary staging based on individualized tumor biology rather than age or clinical features alone. This practice has since been validated by the SOUND trial, published in 2023, which was a randomized controlled trial comparing lumpectomy and breast radiotherapy *without* sentinel node biopsy to lumpectomy and breast radiotherapy *with* sentinel node biopsy among 1405 subjects $\ge$ 18 years of age with clinically negative axilla assessed by axillary ultrasound or negative fine-needle aspiration (FNA).^16^ Median tumor size was 11 mm, and 98.4% of the patients received systemic therapy, whereas 89.3% received whole-breast radiation. During a median follow-up period of 5.7 years, the study found no difference between the two study arms in terms of axillary recurrence (0.4% for the sentinel node group vs 0.7% for the non-sentinel node group), 5-year distant disease-free survival, 5-year disease-free survival, and 5-year OS. In the final analysis, it appears that the FROST investigators and patients were relatively comfortable with omitting sentinel node biopsy in stratum 2 because only 11.4% of the stratum 2 participants actually underwent sentinel node biopsy.

Although 5.7% of the stratum 2 subjects declined both radiotherapy and endocrine therapy, nearly 90% of the stratum 2 subjects received endocrine therapy, radiotherapy, or both. Furthermore, all the stratum 2 subjects had documented clinically negative or FNA-negative nodes at the time of cryoablation, similar to the SOUND trial, which provides evidence supporting the selective omission of sentinel node biopsy for women ages 50 to 59 years treated with cryoablation, radiotherapy, and endocrine therapy for hormone receptor-positive, HER2-negative, clinically node-negative invasive ductal carcinoma. However, with regard to various de-escalation trials, it is important to note that high-level evidence favors the use of endocrine therapy for all patients with hormone receptor-positive disease except those with ultra-low risk genomics.^17^

The FROST trial also provides valuable insights into the safety and efficacy of adjuvant radiotherapy after breast cancer cryoablation. Among the 35 participants in stratum 2, 26 (74.3%) were confirmed to have received adjuvant whole-breast radiotherapy. Although complete radiation treatment records were available for only about half of these individuals, available data documented the delivery of conventional fractionation schedules (4256–6100 cGy in 25 fractions) to all but one patient, who received hypofractionated radiotherapy (4256 cGy in 16 fractions plus a 10-cGy boost). Although the sample size was relatively small, the absence of IBTRs for patients who received adjuvant radiotherapy coupled with the lack of serious adverse events attributable to radiation offers useful information for radiation oncologists counseling patients on post-cryoablation treatment options. These findings suggest that radiotherapy can be safely and effectively incorporated into a cryoablation treatment regimen without increasing the risk of morbidity or compromising patient outcomes.

Despite its strengths, the FROST trial had several important limitations. First, the study closed enrollment before reaching its target accrual of 105 subjects per stratum, potentially limiting the statistical power of the final analyses. Nonetheless, the high response rate (all post-cryoablation core biopsies were negative) and the low incidence of IBTRs (only three events altogether) support the durability of local control. Although the study was not originally powered for IBTR outcomes, the results nonetheless demonstrate high 5-year IBTR-FS rates, with sufficient statistical strength to exceed clinically relevant benchmarks.

The loss of funding also affected other aspects of the study. Specifically, the planned cosmetic outcome assessment, a correlative study initiated at select sites, could not be completed. Furthermore, the study team was unable to comprehensively collect data related to radiation treatment plans. Nonetheless, through the engagement and collaboration of participating co-investigators, we successfully gathered follow-up data on all but two enrolled participants. Key study endpoints, such as response rate, IBTR, invasive IBTR-FS, axillary recurrence, radiotherapy use, endocrine therapy use, and survival, were captured in these subjects, allowing for a robust analysis of the trial’s main objectives.

It is important to acknowledge the potential role of selection bias in the interpretation of the FROST trial results. Patient-related bias likely played a role because the participants in the trial were presumably more engaged in regular breast cancer screening, leading to earlier cancer detection and enrollment of patients with smaller tumors. These individuals also may have been more motivated to avoid breast surgery, lymph node surgery, and radiation therapy, potentially increasing their adherence to recommended adjuvant endocrine therapy.

Physician-related bias may have similarly influenced study enrollment. Although the eligibility criteria permitted tumors up to 20 mm in size, the median tumor size among enrolled participants was only 9 mm, suggesting a preference for selecting patients with smaller lesions. Similarly, although enrollment was open to women age 50 years or older, the median age in stratum 2 was 62 years, suggesting a tendency toward enrolling older patients. Furthermore, although patients with high-grade tumors were eligible for enrollment, most subjects had low- to intermediate-grade cancers, indicating provider bias against enrolling patients with high-grade tumors. Nevertheless, the trial’s multicenter design and the geographic diversity of its participating sites spanning nearly all regions of the United States reflect the broader generalizability of the study’s findings, even in the context of the selection biases.

## Conclusion

The FROST trial builds upon existing data supporting the use of cryoablation as a definitive local therapy for early-stage, hormone receptor-positive, HER2-negative invasive breast cancer. With a 5-year IBTR rate of 3.64%, a 5-year invasive IBTR-FS rate of 97.59%, and a 5-year OS rate of 96.36%, the results demonstrate that cryoablation, where indicated and paired with appropriate adjuvant endocrine therapy and radiotherapy, can be a safe, effective, and well-tolerated alternative to lumpectomy for carefully selected patients.

By incorporating a risk-adapted approach and enrolling women as young as 50 years of age, the FROST trial expands the potential population of patients eligible for cryoablation beyond that studied in earlier trials such as ICE3. The FROST trial also provides valuable insight into evolving care paradigms, including the selective omission of sentinel lymph node biopsy, the safety and efficacy of radiotherapy, and the role of post-ablation biopsy in clinical reassurance and radiologic interpretation.

Despite limitations including early study closure, incomplete correlative data, and potential selection bias, the FROST trial offers compelling evidence supporting the oncologic safety and clinical feasibility of cryoablation as a breast-conserving strategy, especially for women age 70 years or older. The results also support further investigation of the role of cryoablation in the management of younger patients. The FROST trial adds to the growing body of literature advocating for de-escalation of breast surgery for selected patients and underscores the need for continued evaluation of cryoablation in both clinical practice and future trials, especially in patients for whom conventional surgery may be less desirable or less feasible.

## Supplementary Information

Below is the link to the electronic supplementary material.

Supplementary file1 (DOCX 1486 kb)
