# Supplementary material for: Cryoablation: A Minimally Invasive Alternative for Early-Stage Breast Cancer: 6-Year Outcomes of the FROST Clinical Trial
Source: Ann Surg Oncol. 2026 Jan 15;33(4):3374–82. doi: 10.1245/s10434-025-18991-2 (PMC12982317; doi:10.1245/s10434-025-18991-2)
Supplement: Supplementary file 1 — Supplementary file1 (DOCX 1486 kb) [file 10434_2025_18991_MOESM1_ESM.docx]

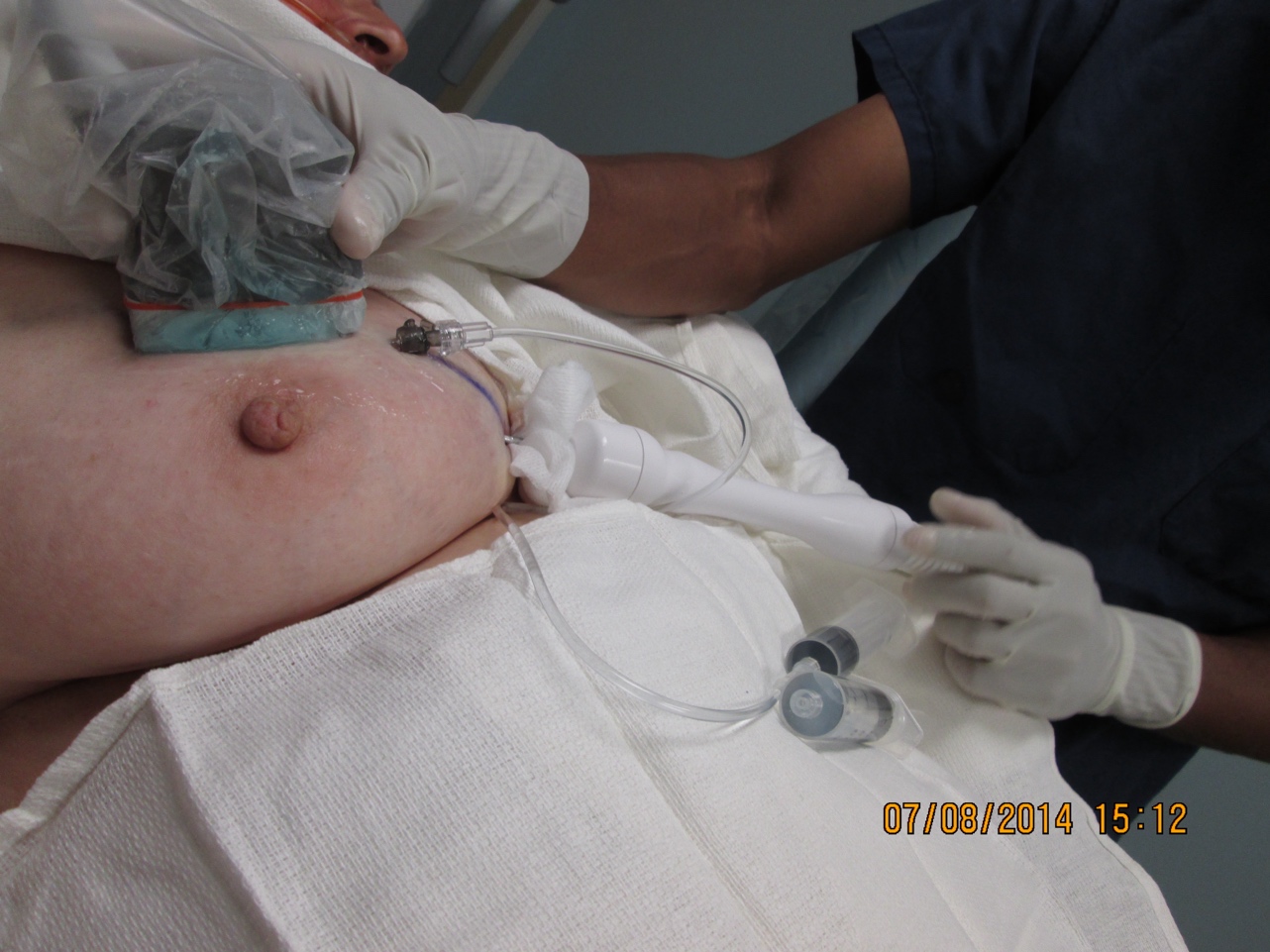


Supplemental Image 1 shows an office-based cryoablation procedure performed under ultrasound guidance.


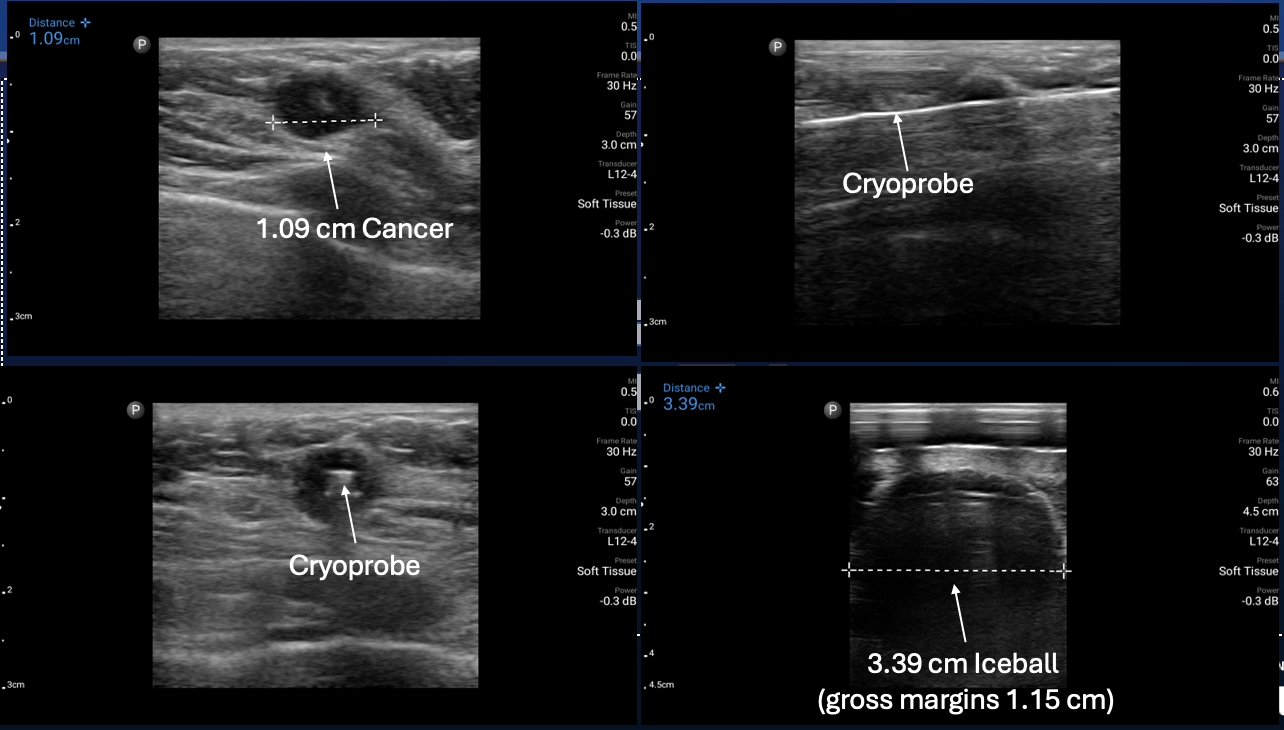


Cancer

C

D

B

A

A

Supplemental Image 2. A. Ultrasound image showing a 1.09 cm cancer. B. Ultrasound image showing cryoprobe traversing central axis of cancer (longitudinal view). C. Ultrasound image showing cryoprobe traversing central axis of cancer (transverse view). D. Ultrasound image of iceball with 3.39 cm transverse diameter, achieving 1.15 cm transverse ablation margins (3.39 cm iceball diameter minus 1.09 cm tumor diameter = 2.3 cm divided by 2 = 1.15 cm ablation margins).

| **Study Site** | **Location** |
| --- | --- |
| Adventist Health Glendale Medical Center | Glendale, CA |
| Ascension Crittenton Hospital | Rochester, MI |
| Brown University | Providence, RI |
| City of Hope | Duarte, CA |
| Diagnostic Center for Women | Miami, FL |
| Epic Care | Emeryville, CA |
| Knoxville Comprehensive Breast Care | Knoxville, TN |
| Lankenau Medical Center | Wynnewood, PA |
| Naples Community Hospital | Naples, FL |
| Ogden Regional Medical Center | Ogden, UT |
| Southwest Cancer Center | Lubbock, TX |
| 90120 Surgery Medical Center | Beverly Hills, CA |

Supplemental Table 1. Study sites and locations where trial participants were enrolled.

| 2 Weeks post-cryoablation | | | | |
| --- | --- | --- | --- | --- |
|  | Grade 0 | Grade 1 | Grade 2 | Grade 3 |
| Pain: Breast | 62.0% | 31.5% | 6.5% | 0.0% |
| Dermatology: Bruising/Hematoma | 76.9% | 22.2% | 0.9% | 0.0% |
| Infection: Unknown ANC - Wound) | 93.5% | 4.6% | 1.9% | 0.0% |
| Localized Edema: Trunk/Genital) | 90.7% | 7.4% | 1.9% | 0.0% |
| Post Operative Hemorrhage | 97.2% | 2.8% | 0.0% | 0.0% |
| Skin Ulceration: Breast | 97.2% | 2.8% | 0.0% | 0.0% |
| Dizziness | 97.2% | 2.8% | 0.0% | 0.0% |
| Seroma | 100.0% | 0.0% | 0.0% | 0.0% |
| Nausea | 100.0% | 0.0% | 0.0% | 0.0% |
| Pain: Head/Headache | 100.0% | 0.0% | 0.0% | 0.0% |
| Breast Nipple Deformity | 100.0% | 0.0% | 0.0% | 0.0% |
| Anaphylactic Reaction | 100.0% | 0.0% | 0.0% | 0.0% |
| Allergic Reaction | 100.0% | 0.0% | 0.0% | 0.0% |

| 6-Months post-cryoablation | | | | |
| --- | --- | --- | --- | --- |
|  | Grade 0 | Grade 1 | Grade 2 | Grade 3 |
| Pain: Breast | 76.3% | 13.2% | 10.5% | 0.0% |
| Dermatology: Bruising/Hematoma | 92.1% | 7.9% | 0.0% | 0.0% |
| Infection: Unknown ANC - Wound) | 100.0% | 0.0% | 0.0% | 0.0% |
| Localized Edema: Trunk/Genital) | 92.1% | 7.9% | 0.0% | 0.0% |
| Post Operative Hemorrhage | 100.0% | 0.0% | 0.0% | 0.0% |
| Skin Ulceration: Breast | 100.0% | 0.0% | 0.0% | 0.0% |
| Dizziness | 92.1% | 0.0% | 7.9% | 0.0% |
| Seroma | 100.0% | 0.0% | 0.0% | 0.0% |
| Nausea | 94.7% | 5.3% | 0.0% | 0.0% |
| Pain: Head/Headache | 94.7% | 0.0% | 5.3% | 0.0% |
| Breast Nipple Deformity | 100.0% | 0.0% | 0.0% | 0.0% |
| Anaphylactic Reaction | 100.0% | 0.0% | 0.0% | 0.0% |
| Allergic Reaction | 100.0% | 0.0% | 0.0% | 0.0% |

Supplemental Table 2. A. Adverse events and Grades assessed 2 weeks post- cryoablation. B. Adverse events and Grades assessed 6 months post-cryoablation.
